# Effect of VAChT reduction on lung alterations induced by exposure to iron particles in an asthma model

**DOI:** 10.1186/s12950-024-00399-6

**Published:** 2024-07-03

**Authors:** Tabata Maruyama dos Santos, Renato Fraga Righetti, Leandro do Nascimento Camargo, Edna Aparecida Leick, Silvia Fukuzaki, Elaine Cristina de Campos, Thiago Tafarel Galli, Beatriz Mangueira Saraiva-Romanholo, Luana Laura Sales da Silva, Jéssica Anastácia Silva Barbosa, Juliana Morelli Lopes Gonçalves João, Carla Máximo Prado, Bianca Goulart de Rezende, Christine Laure Marie Bourotte, Fernanda Degobbi Tenorio Quirino dos Santos Lopes, Milton de Arruda Martins, Isabela M. Bensenor, João Vitor de Oliveira Cirillo, Suellen Karoline Moreira Bezerra, Fabio José Alencar Silva, Marcela Souza Lima Paulo, Paulo A. Lotufo, Iolanda de Fátima Lopes Calvo Tibério

**Affiliations:** 1 Faculdade de Medicina FMUSP, Universidade de Sao Paulo, São Paulo, SP Brazil; 2 Hospital Sírio Libanês, São Paulo, SP Brazil; 3 Hospital Alemão Oswaldo Cruz, São Paulo, Brazil; 4 University City of São Paulo (UNICID), São Paulo, Brazil; 5 Department of Biosciences, Universidade Federal de São Paulo, Santos, Brazil; 6 Escola Superior de Ciências da Santa Casa de Misericórdia de Vitória, Vitória, Brazil; 7 Instituto de Geociências, Universidade de São Paulo, São Paulo, Brasil

**Keywords:** Asthma, Acetylcholine, Particulate matter, Pollution, Inflammation

## Abstract

**Introduction:**

Pollution harms the health of people with asthma. The effect of the anti-inflammatory cholinergic pathway in chronic allergic inflammation associated to pollution is poorly understood.

**Methods:**

One hundred eight animals were divided into 18 groups (6 animals). Groups included: wild type mice (WT), genetically modified with reduced VAChT (VAChTKD), and those sensitized with ovalbumin (VAChTKDA), exposed to metal powder due to iron pelletizing in mining company (Local1) or 3.21 miles away from a mining company (Local2) in their locations for 2 weeks during summer and winter seasons. It was analyzed for hyperresponsivity, inflammation, remodeling, oxidative stress responses and the cholinergic system.

**Results:**

During summer, animals without changes in the cholinergic system revealed that Local1 exposure increased the hyperresponsiveness (%Rrs, %Raw), and inflammation (IL-17) relative to vivarium animals, while animals exposed to Local2 also exhibited elevated IL-17. During winter, animals without changes in the cholinergic system revealed that Local2 exposure increased the hyperresponsiveness (%Rrs) relative to vivarium animals. Comparing the exposure local of these animals during summer, animals exposed to Local1 showed elevated %Rrs, Raw, and IL-5 compared to Local 2, while in winter, Local2 exposure led to more IL-17 than Local1. Animals with VAChT attenuation displayed increased %Rrs, NFkappaB, IL-5, and IL-13 but reduced alpha-7 compared to animals without changes in the cholinergic system WT. Animals with VAChT attenuation and asthma showed increased the hyperresponsiveness, all inflammatory markers, remodeling and oxidative stress compared to animals without chronic lung inflammation. Exposure to Local1 exacerbated the hyperresponsiveness, oxidative stressand inflammation in animals with VAChT attenuation associated asthma, while Local2 exposure led to increased inflammation, remodeling and oxidative stress.

**Conclusions:**

Reduced cholinergic signaling amplifies lung inflammation in a model of chronic allergic lung inflammation. Furthermore, when associated with pollution, it can aggravate specific responses related to inflammation**, **oxidative stress, and remodeling**.**

**Supplementary Information:**

The online version contains supplementary material available at 10.1186/s12950-024-00399-6.

## Introduction

Particulate matter (PM) contains toxic components generated from industrial products, vehicular emissions, coal combustion, sea salt and dust [1]. These particles may include sulfates, nitrates, and chemical elements such as iron, silica, and aluminum [2]. Exposure to these pollutants could induce respiratory symptoms, asthma exacerbations, hospitalizations, and a high risk of premature deaths [3].

The size of PM is linked to health risks ine particles can penetrate into the bronchioles and alveoli, remaining suspended in the atmosphere for extended periods, thereby increasing the likehood of inhalation [4, 5].

To observe the impact of pollution from iron dust, the study focused on the Greater Vitória Metropolitan Region (RMGV) in the State of Espírito Santo, Brazil Covering an area of 1448 miles, this region is characterized bykey urban and industrial development centers. With a population of 2.0 million inhabitants [6], its topographyvaries from a coastal plain to hills, and its proximity to the ocean influences weather conditions, favoring the dispersion of certain pollutants. During winter, unfavorable conditions—such as dry weather, reduced soil moisture, and lower evaporation rates—contribute to deteriorated air quality due to the formation of convective clouds and reduced turbulent movements [7].

Vitória faces emissions from three primary sources of air pollutants: vehicles, the mining and steel industry, and port and airport operations [8]. In the area, approximately 70% of PM emissions in the region originate from stem mill activities and pelletizing process within the industrial complex [9].

Asthma, a heterogeneous disease [10], is characterized by bronchial hyperresponsiveness, lung inflammation, airway remodeling response, and airway obstruction [11] and air pollution has a negative impact on outcomes [12].

In the pulmonary airways, acetylcholine (Ach) serves as the parasympathetic neurotransmitter and acts as the main mediator of the anti-inflammatory cholinergic system [13]. The vesicular acetylcholine transporter (VAChT) is essential for releasing ACh into the synaptic cleft, with the amount of ACh released directly proportional to VAChT levels [14]. Once released, ACh stimulates both peripheral and central muscarinic (mAChR) and nicotinic (nAChR) receptors [15, 16].

While studies have implicated ACh in mAChR involvement in airway inflammation and remodeling in asthmatic patients and animal models [17–19], reduced VAChT levels create a pro-inflammatory environment in the lung. This suggests that endogenous ACh deficiency contributes to the pathogenesis of chronic allergic airway inflammation and may lead to changes in nAChR expression [20, 21].

Knowing that the decrease in VAChT can promote worsening of lung inflammation, we intend to study the effects of iron dust and pollution in mice with modification of the VAChT receptor and evaluate whether pollution and exposure to iron dust potentiate chronic allergic lung inflammation in animals with a modified VAChT receptor. Our assessment will include lung mechanics, inflammatory markers, remodeling, and responses to oxidative stress during summer and winter seasons.

## Materials and methods

### Ethical approval and animal care

This study, involving a total of 108 animals, received approval from the Research Ethics Committee of the Hospital das Clínicas, Faculty of Medicine, University of São Paulo (approval number: 1191/2018). All procedures were conducted in strict accordance with the Guidelines for the Care and Use of Laboratory Animals published by the National Institute of Health.

### Experimental protocols

Two experimental protocols were performed, as detailed below.

#### Protocol experimental 1 (WT animals X VAChT KD)

To explore the effects of iron dust and pollution on mice with modified VACHT expression, this protocol was carried out with heterozygous mice intercrossed to generate VAChT KD and wild-type (WT) mice.

VAChT KD mice were produced as described by Prado et al. (2006) [22]. The VAChT mice was criated several years ago to study initially the effects of VAChT reduction in brain. A lot of studies have been published with these animals [20, 21, 23].

Studies using the lung from VAChT KD animals, elucidated that VAChT is reduced not only in nervous system but also in lung from KD mice, Pinheiro et al., 2015 showed a reduction in VAChT mRNA and protein expression in spinal cord and in lung, one important phenotype from the VAChTKD is the reduction of body weight [21]. We performed body weight analysis in the animals in the present study and clearly showed a reduction in weight in VAChT KD mice. The weight is given below in mean and standard error: VAChT KD mice (19.2 ± 0.6) and WT mice (21.6 ± 0.6), *p* < 0.05.

These mice exhibit a 65%-70% reduction in vesicular acetylcholine transporter (VAChT) [20, 22], resulting in a proportional decrease in acetylcholine (ACh) release within the lungs [21].

Experimental Group 1:

- WT (wild-type mice), these animals will remain in the vivarium without exposure;
- WT Local1 (wild-type mice exposed to metal powder due to pelletizing iron ore at a mining company);
- WT Local2 (wild-type mice exposed to metal powder 3.21 mi away from a mining company);
- VAChT KD (mice with reduced VAChT expression), these animals will remain in the vivarium without exposure;
- VAChT KD-Local1 (mice with reduced VAChT expression exposed to metal powder due to pelletizing iron ore at a mining company);
- VAChT KD-Local2 (mice with reduced VAChT expression exposed to metal powder 3.21 mi away from a mining company).

The animals were exposed to pollution from iron dust in summer and winter.

#### Protocol experimental 2 (VAChT KD animals X VAChT KD with chronic allergic airway inflammation)

To investigate the effects of VAChT deficiency on OVA-induced airway inflammation associated with exposure to iron dust, we implemented the following protocol:

Experimental Group 2:

- VAChT KD (mice with reduced VAChT expression), these animals will remain in the vivarium without exposure;
- VAChT KD-Local1 (mice with reduced VAChT expression exposed to metal powder due to pelletizing iron ore at a mining company);
- VAChT KD-Local2 (mice with reduced VAChT expression exposed to metal powder 3.21 mi away from a mining company).
- VAChT KDA (mice with reduced VAChT expression sensitized with OVA), these animals will remain in the vivarium without exposure;
- VAChT KDA-Local1 (mice with reduced VAChT expression sensitized with OVA and exposed to metal powder due to pelletizing iron ore at a mining company);
- VAChT KDA-Local2 (mice with reduced VAChT expression sensitized with OVA and exposed to metal powder 3.21 mi away from a mining company).

The animals were exposed to pollution in summer and winter.

#### Chronic allergic airway inflammation protocol

The protocol for sensitizing mice and inducing pulmonary inflammation using ovalbumin lasted 29 days. On Days 1 and 14, the mice received an intraperitoneal injection of a solution containing 50 mg of ovalbumin (Sigma-Aldrich) and 6 mg of aluminum hydroxide (Alumen, Pepsamar, Sanofi-Synthelabo SA, Rio de Janeiro, Brazil) in a total volume of 0.2 ml. On Days 22, 24, 26, and 28, the mice were placed in an acrylic box (30 × 15 × 20 cm) connected to an ultrasonic nebulizer (US-1000, ICEL, São Paulo, Brazil) and exposed to an aerosol of ovalbumin diluted in saline (0.9% NaCl) at a concentration of 10 mg/ml (1%) for 30 min.

### Environmental exposure protocol

**Fig. 1:**
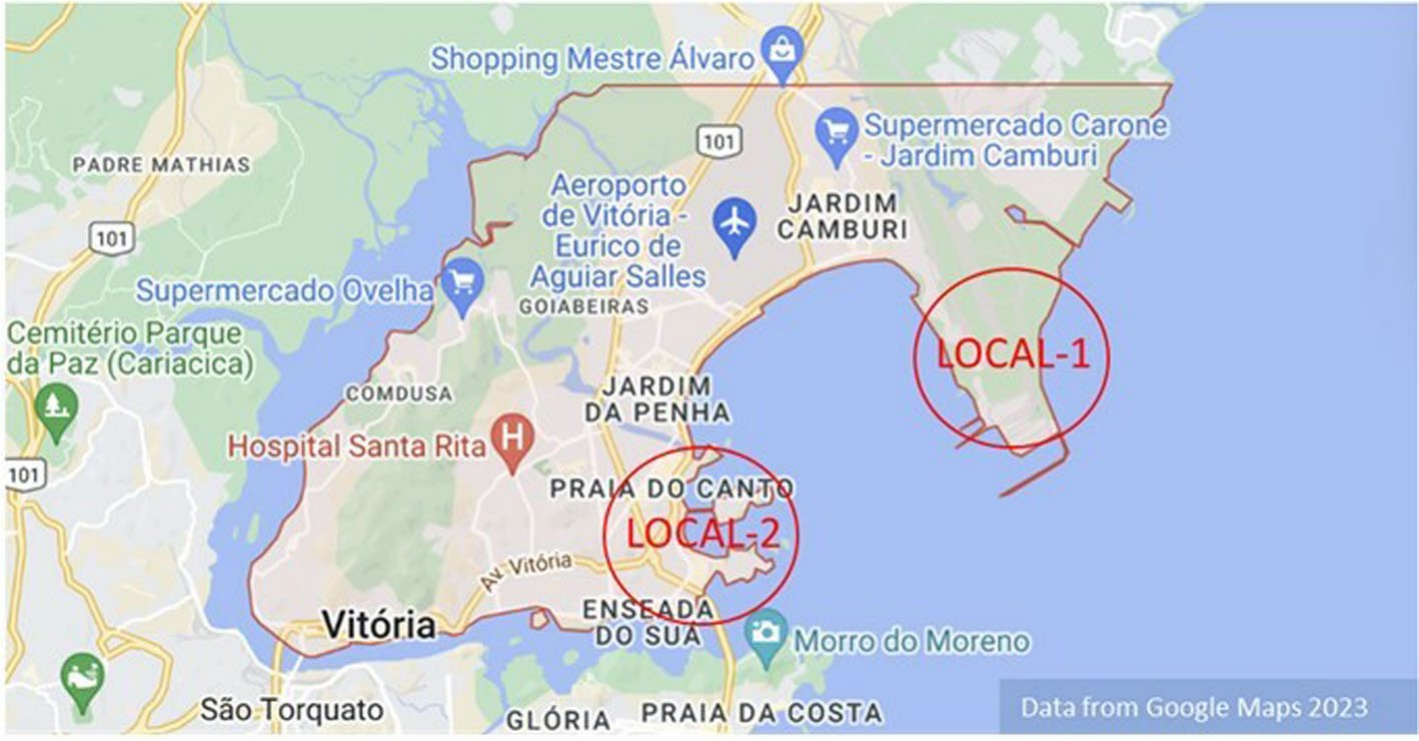
Local 1 (animals exposed to iron dust pollution inside a mining company) and Local 2 (animals exposed to iron dust pollution 3.21 mi from the mining company)

The animals were exposed at two locations in Vitória, in the state of Espírito Santos, Brazil, and a vivarium located in São Paulo – SP, Brazil. The exhibition points in Vitória were Local 1 and Local 2 (Fig. 1).

- Vivarium: closed environment, appropriate temperature, with filtered air.
- Local 1: Located inside the mining company, one of the main processes generated in this pelletizing company.
- Local 2: Situated on the top floor of a building in Ilha do Boi, approximately 3.21 miles from the mining company. This area is considered prime in the city, with many hotels.

**Fig. 2:**
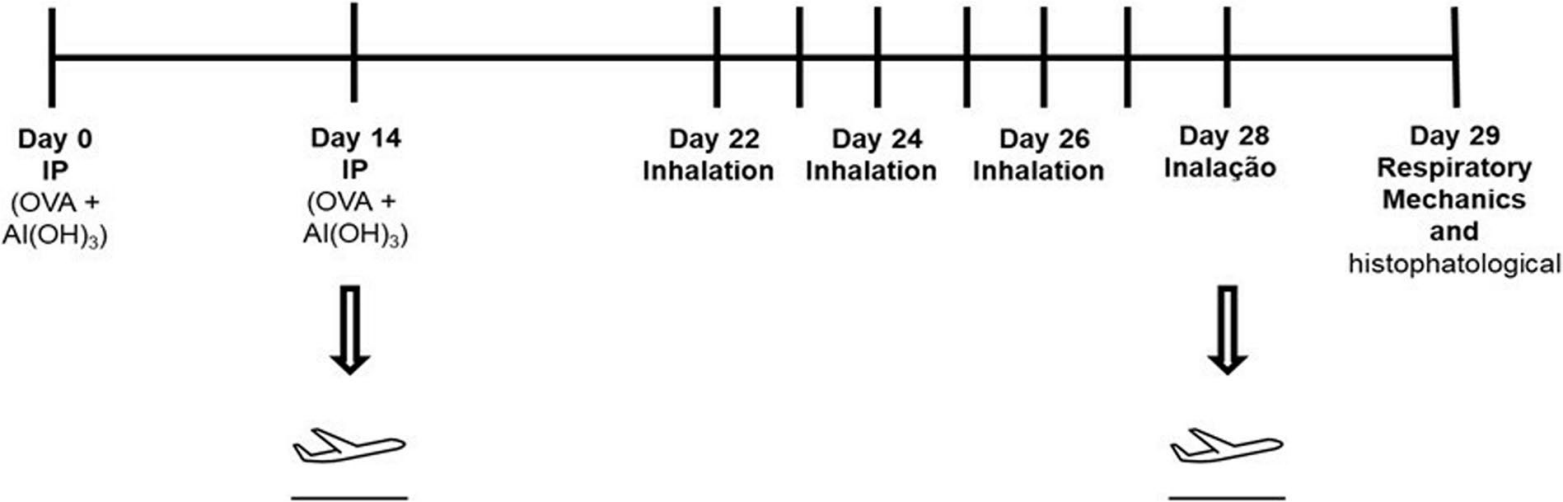
Experimental Protocol 2: Comparative analyses between VAChT KD animals and VAChT KDA animals

The animals exhibited in Vitória were kept in a protected environment, breathing the ambient air of the city; the rooms in which they stayed remained with the windows open. After two weeks in Vitória, the animals were returned to the city of São Paulo—SP for evaluations (Fig. 2).

#### Particulate characterization

Particulate analysis and characterization were conducted in parallel under the responsibility of Prof. Christine Bourotte (Cidade Universitária, São Paulo-SP).

Sampling occurred during 24-h intervals during December 2018 (summer period), and June 2019 to the first week of August 2019 (winter period) at both experimental sites (local 1 and local 2). The summer sampling campaign totalized 18 days and winter campaign also totaled 36 days.

During summer sampling period, cumulative precipitation was 76.4 mm, mean temperature was 26 ± 3.5°Celsius and air relative humidity was 74.6 ± 14.4%. During the winter sampling period, cumulative precipitation was 52.6 mm, mean temperature was 20.6 ± 3.5° Celsius and air relative humidity was 78,9 ± 16.9%.

The fine fraction (d < 2.5 μm) and coarse fraction (2.5–10 μm) of the particulate matter were collected using a dichotomous sampler with a Stacked Unit Filter (SFU) for 47 mm diameter filters, as described by Hopke et al. (1997) [24]. Polycarbonate membrane filters with 8.0 μm and 0.45 μm pore diameter were used to collect the coarse fraction and the fine fraction, respectively. The PM_10_ fraction (< 10 μm) corresponds to the sum of the coarse and fine fractions.

Elemental analysis (Al, Si, P, S, Cl, K, Ca, Ti, V, Cr, Mn, Fe, Ni, Zn, Br, and Pb) was performed by EDXRF—Spectrometer EDX 700HS; Shimadzu Corporation [25]. The filter was submitted to EDXRF, and spectra accumulated for 900 s under the following conditions: Al filter, vacuum as X-ray path, 10-mm diameter collimator, 10–20 keV energy range, 50 kV tube voltage, an Rh X-ray tube, and a Si (Li) detector. The spectra were reduced with WinQXAS software. Blank filters were also analyzed to evaluate elements by EDXRF and discounted in samples.

#### Particulate matter concentrations and common elements in the analysis

During both summer and winter, mean PM concentrations at Local 1 were higher than those at Local 2. According to unpublished data, the coarse fraction represented approximately 80% of PM_10_. During summer period, PM_10_ concentrations were 164 ± 112 μg.m^−3^ and 33.6 ± 12.1 μg.m^−3^ at local 1 and local 2, respectively. During winter period, the PM_10_ concentrations were 51.2 ± 27.8 μg.m^−3^ and 36.6 ± 13.0 μg.m-3 at local 1 and local 2, respectively.

The most common elements identified in the analysis were chloride (Cl), iron (Fe), and sulfur (S). Iron concentrations were higher in local 1 than in local 2, while chlorine concentrations were higher in local 2 than in local 1 (see Table 1).

**Table 1:** Elemental concentration in fine and coarse PM fraction in both sampling station and sampling period. (*n* = number; Std. = standard deviation; Min. = minimum value; Max. = maximum value)

	**Local 1**									
	Summer					Winter				
**Coarse PM**	***n***	***Mean***	***Std***	***Min***	***Max***	***n***	***Mean***	***Std***	***Min***	***Max***
		*(µg.m*^*−3*^*)*					*(µg.m*^*−3*^*)*			
**Cl**	16	2.260	1.255	0.625	5.309	28	2.146	1.739	0.357	6.632
**Fe**	16	40.55	33.74	2.910	116.2	28	9.931	8.049	1.947	31.72
**Na**	12	0.584	0.590	0.044	1.686	27	0.393	0.324	0.050	1.252
**S**	16	1.241	0.828	0.420	3.014	28	0.396	0.207	0.167	0.875
**Fine PM**										
**Cl**	16	0.227	0.253	0.012	0.796	27	0.191	0.182	0.022	0.628
**Fe**	16	5.504	3.544	0.598	12.62	27	1.691	1.456	0.056	5.880
**Na**	12	0.323	0.324	0.029	0.925	20	0.106	0.091	0.010	0.342
**S**	16	1.319	0.653	0.328	2.234	27	0.361	0.259	0.056	1.124
	**Local 2**									
	Summer					Winter				
**Coarse PM**	***n***	***Mean***	***Std***	***Min***	***Max***	***n***	***Mean***	***Std***	***Min***	***Max***
		*(µg.m*^*−3*^*)*					*(µg.m*^*−3*^*)*			
Cl	18	5.933	1.606	2.703	9,042	18	4.530	1.461	2.448	7.128
Fe	18	3.35	3.001	0.260	9,341	18	1.209	0.871	0.217	3.361
Na	18	1.336	0.354	0.665	2,350	18	0.907	0.278	0.459	1.484
S	18	0.812	0.257	0.373	1,396	18	0.601	0.166	0.366	0.907
**Fine PM**										
Cl	17	0.160	0.187	0.004	0.746	18	0.213	0.130	0.035	0.486
Fe	17	0.172	0.208	0.022	0.749	18	0.354	0.345	0.042	1.146
Na	14	0.131	0.096	0.023	0.410	18	0.119	0.078	0.010	0.267
S	17	0.324	0.303	0.032	1.288	18	0.350	0.224	0.088	0.941

### Measurement of exhaled nitric oxide

On the 29th day of the experimental protocol, the animals were anesthetized with an intraperitoneal injection of thiopental (50 mg/kg) and underwent tracheostomy. To obtain exhaled NO, after stabilizing the animal on the ventilator, a collection balloon was adapted to the ventilator's expiratory outlet for 10 min [26, 27]. Exhaled NO was measured using the chemiluminescence technique with a rapid response analyzer (Sievers Instruments, Boulder, USA) that was calibrated with a certified source of 47 parts per billion (ppb). NO filter (Sievers Instruments, Boulder, USA) was attached to the inspiratory limb of the ventilator to avoid environmental contamination and to keep inspired air free of nitric oxide before each measurement.

#### Evaluation of lung hyperresponsiveness

The animals were connected to a mechanical ventilation (FlexiVent, Scireq, Montreal, Canada) and ventilated with a tidal volume of 10 ml/kg, respiratory rate of 120 cycles/minutes and sinusoidal inspiratory flow curve. To eliminate ventilatory effort, the animals received an intraperitoneal injection of pancuronium (0.2 mg/kg) [11, 21]. Pressure values were generated, and airway impedance (pressure/flow) was calculated as a function of the different frequencies produced [28].

After 30 s of ventilation, the basal measures of resistance and elastance of the animals were performed. The challenge was performed with inhalation of methacholine at doses of 3, 30, and 300 mg/ml in the first 30 s, and first, second, and third minutes. Measurements were then obtained for the following parameters of the respiratory system: resistance of the respiratory system (Rrs), elastance of the respiratory system (Ers), airway resistance (Raw), pulmonary tissue resistance (Gtis), and pulmonary tissue elastance (Htis) were obtained. The maximum response of each measure of the respiratory system was used for the study.

At the end of the evaluation, the mice were euthanized by intraperitoneal injection of sodium pentobarbital (50 mg/kg i.p.) and immediately heparinized (1000 IU) intravenously and the abdominal aorta and vena cava were sectioned to euthanize. The lungs were removed *in bloc* with the heart for morphometric studies and histological/histochemical analyses [29].

### lmunohistochemistry

Immunohistochemistry was performed as described by Santos et al. [30]. Immunohistochemical data are in supplementary material. Eosinophil counts were assessed using hematoxylin–eosin staining. Collagen fibers were marked using Picro-Sirius [30].

### Morphometric analysis

The technique of counting points was used with the reticle of 100 points and 50 lines attached to the microscope eyepiece [30], with a total area of 10^4^ µm^2^. To quantify airway-positive cells, the reticulum was attached to the base of the epithelium. The ratio between the number of cells in a given area and the number of points that coincided with the peribronchial airway area was determined. We evaluated 3–4 airways per animal [30]. Analyses were performed at 1000 × magnification [31].

### Image analysis

For the analysis of collagen fibers and 8-PGF-2α, we employed image analysis. Images were captured using a Leica DM2500 microscope (Leica Microsystems, Wetzlar, Germany) equipped with a coupled camera that transmitted the images to a computer. Four airways were quantified per animal. Image analysis was performed using Image-Pro Plus 4.5 software (NIH, Bethesda, MD, United States) [31]. The volume fractions of these markers were expressed as a percentage of the total area [31, 32].

### Statistical analysis

Statistical analysis was performed using SigmaStat 11.0 (SPSS Inc., Chicago, IL). The one-way analysis of variance test was used according to the *Holm‒Sidak* method for group comparisons. In the comparative analysis between even groups, *the rank sum test* was performed. The results were expressed in the form of medium and standard error and displayed in a bar graph. *p* < 0.05 was considered statistically significant for all analyses.

## Results

Below is the presentation of the results referring to protocol 1 (analysis of WT animals compared to animals with reduced VAChT and effects of exposure to the sites on these animals) and presentation of the results referring to protocol 2 (analysis of animals with reduced VAChT compared to animals with decreased VAChT associated with chronic allergic lung inflammation and effects of site exposure in these animals).

### Experimental protocol 1 (Fig. 3)

**Fig. 3:**
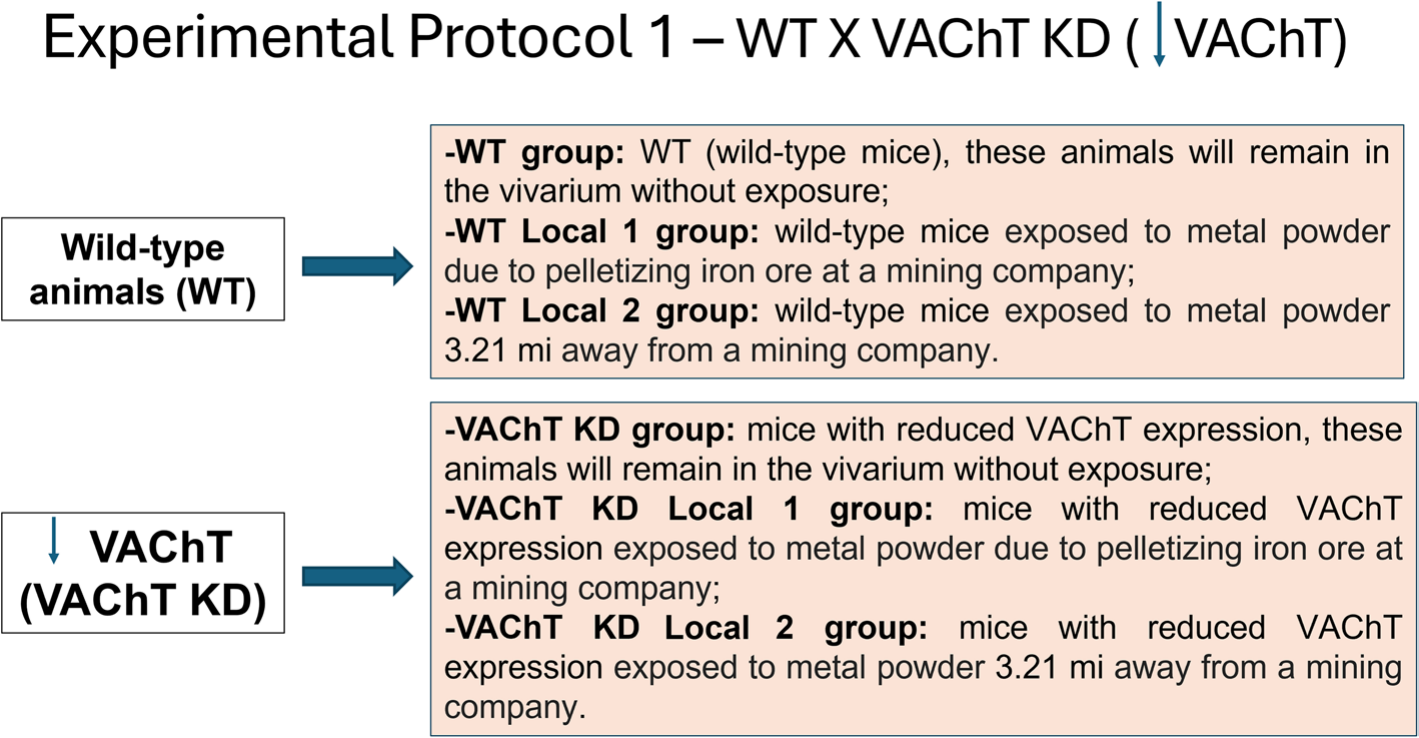
Experimental Protocol 1: Comparative analysis between WT animals and VAChT KD animals

#### Hyperresponsiveness

In relation to hyperresponsivity, WT animals exposed during the summer to Local 1 showed an increase in %Rrs and %Raw compared to WT animals that remained in the vivarium (*p* < 0.05) and WT animals that remained in Local 2 (*p* < 0.05). In winter, WT animals exposed to Local 2 demonstrated an increase in %Rrs compared to WT animals that remained in the vivarium (*p* < 0.05).

In the genetically modified animals with a decrease in VAChT, we observed that in the summer, there was an increase in %Rrs in animals exposed to Local 1 compared to animals exposed to Local 2 (*p* < 0.05). In Local 2, there was an increase in %Ers and %Htis in these animals compared to animals with a decrease in VAChT exposed to Local 1 (*p* < 0.05) (Table 2).

**Table 2:** Analysis of hyperresponsiveness, cholinergic system, inflammation, and remodeling (Experimental Protocol 1)

Hyperresponsiveness						
** Summer**	**WT**	**WT-Local 1**	**WT-Local 2**	**VAChT KD**	**VAChT KD-Local1**	**VAChT KD-Local2**
%Rrs	35.9 ± 8.7	**109.5 ± 15.5**^**€,ϒ**^	73.4 ± 10.06	**70.5 ± 12.5**^**€**^	**125.2 ± 33.2**^**#**^	50.3 ± 12.0
%Ers	30.8 ± 7.4	68.7 ± 13.6	41.1 ± 8.2	17.2 ± 3.7	39.4 ± 4	46.2 ± 7.3^*^
%Raw	80.3 ± 10.6	**169.9 ± 32.8**^**€,ϒ**^	81.1 ± 16.09	102.9 ± 20.0	177.2 ± 55.7	94.6 ± 29.1
%Gtis	35.2 ± 6.5	48.2 ± 9.3	25.1 ± 5.7	44.1 ± 10,6	68.8 ± 21.5	23.1 ± 3.7
%Htis	26.7 ± 6.9	45.05 ± 9.1	45.3 ± 11.3	22.4 ± 4.4	34.2 ± 11.4	58.4 ± 9.3*
** Winter**	**WT**	**WT-Local 1**	**WT-Local 2**	**VAChT KD**	**VAChT KD-Local1**	**VAChT KD-Local2**
%Rrs	29.0 ± 6.4	70.4 ± 26.7	**114.6 ± 19.6**^**€**^	**70.6 ± 12.6**^**€**^	79.0 ± 26.4	90.2 ± 31.2
%Ers	25.1 ± 5.9	45.9 ± 6.04	40.5 ± 4.8	21.5 ± 7.6	38.9 ± 3.9	24.8 ± 5.6
%Raw	72.9 ± 9.2	69.7 ± 32.3	133.1 ± 24.6	102.8 ± 24.6	81.7 ± 16.0	145.1 ± 51.5
%Gtis	30.8 ± 5,8	57.2 ± 16.9	35.4 ± 6.9	43.0 ± 11,2	24.6 ± 7.8	33.2 ± 13.4
%Htis	21.6 ± 5.8	30.7 ± 2.4	28.6 ± 5.5	20.4 ± 4.8	35.3 ± 6.3	17.3 ± 6.0
**Cholinergic System**						
** Summer**	**WT**	**WT-Local1**	**WT-Local2**	**VAChT KD**	**VAChT-KD Local1**	**VAChT-KD Local2**
AChR α-7 (cells/10^4^μm2)	3.9 ± 0.8*	3.6 ± 0.9**	2.1 ± 0.5	1.5 ± 0.3	1.1 ± 0.3	1.0 ± 0.3
** Winter**	**WT**	**WT-Local1**	**WT-Local2**	**VAChT KD**	**VAChT-KD Local1**	**VAChT-KD Local2**
AChR α-7 (cells/10^4^μm2)	4.7 ± 1.0*	9.4 ± 1.9	**8.1 ± 1.2**^**#**^	1.4 ± 0.4	6.4 ± 1.1*^,#^	3.5 ± 0.8
**Inflammation**						
** Summer**	**WT**	**WT-Local1**	**WT-Local2**	**VAChT KD**	**VAChT-KD Local1**	**VAChT-KD Local2**
NFkappaB (cells/10^4^μm2)	0,9 ± 0,1	0.6 ± 0.1	1.1 ± 0.2	**9.5 ± 0.9**^**€**^	**6.4 ± 0.8**^**Ϟ**^	**7.4 ± 1.1**^**ϒ**^
IL-5 (cells/10^4^μm^2^)	2.8 ± 0.5	**4.7 ± 0.7**^**ϒ**^	2.0 ± 0.4	**3.2 ± 3.8**^**€**^	6.3 ± 0.8^*,#,Ϟ^	**4.03 ± 0.4**^**ϒ**^
IL-13 (cells/10^4^μm^2^)	1.8 ± 0.5	2.5 ± 0.4	2.8 ± 0.5	**2.9 ± 0.3**^**€**^	**3.4 ± 0.4**^**Ϟ**^	**3.3 ± 0.4**^**ϒ**^
IL-17 (cells/10^4^μm^2^)	2.2 ± 0.3	2,5 ± 0,2*	**4.5 ± 0.5**^**€,Ϟ**^	3.2 ± 0.4	2.0 ± 0.5	5.2 ± 0.8*,**
** Winter**	**WT**	**WT-Local1**	**WT-Local2**	**VAChT KD**	**VAChT-KD Local1**	**VAChT-KD Local2**
NFkappaB (cells/10^4^μm2)	0.8 ± 0.1	0.4 ± 0.1	0.9 ± 0.2	**8.9 ± 1.0**^**€**^	16.3 ± 1.7*^,#,Ϟ^	**8.9 ± 1.2**^**ϒ**^
IL-5 (cells/10^4^μm^2^)	2.6 ± 0.6	3.0 ± 0.6	2.7 ± 0.4	**3.2 ± 0.3**^**€**^	5.9 ± 5.7*^,#,Ϟ^	**3.2 ± 3.9**^**ϒ**^
IL-13 (cells/10^4^μm^2^)	1.9 ± 0.5	3.4 ± 0.6	2.4 ± 0.4	**2.6 ± 0.3**^**€**^	**3.7 ± 0.4**^**Ϟ**^	2.7 ± 0.4
IL-17 (cells/10^4^μm^2^)	2.3 ± 0.3	**4.1 ± 0.4**^**€**^	3.1 ± 0.3	3.1 ± 0.5	4.2 ± 0.7	3.6 ± 0.7
**Remodelling**						
** Summer**	**WT**	**WT-Local1**	**WT-Local2**	**VAChT KD**	**VAChT-KD Local1**	**VAChT-KD Local2**
TGF-β (cells/10^4^μm^2^)	3.8 ± 0.7	3.5 ± 0.6	3.0 ± 0.6	4.5 ± 0.9	5.6 ± 1.0	**5.8 ± 0.9**^**ϒ**^
** Winter**	**WT**	**WT-Local1**	**WT-Local2**	**VAChT KD**	**VAChT-KD Local1**	**VAChT-KD Local2**
TGF-β (cells/10^4^μm^2^)	3.7 ± 0.7	5.7 ± 1.3	3.6 ± 0.6	3.9 ± 0.8	**7.6 ± 1.2**^**Ϟ**^	6.3 ± 1.1

^*^p < 0.05 compared to VAChT KD, **p < 0.05 compared to VAChT KD-Local1, #*p* < 0.05 compared to VAChT KD-Local2, ^€^*p* < 0.05 compared to WT, ^Ϟ^*p* < 0.05 compared to WT-Local1, ^ϒ^*p* < 0.05 compared to WT-Local2

#### Cholinergic system

Regarding the analysis of AChR α-7-, we observed a decrease in α-7 in VAChT KD animals compared to WT animals (*p* < 0.05).

Comparing the VAChT KD animals, we found that there was an increase in α-7 in the VAChT KD Local-1 group compared to the VAChT KD and VAChT Local-2 group (*p* < 0.05) (Table 2).

#### Inflammation

There was an increase in NF-KappaB-, IL-5-, IL-13- positive cells in VAChT KD group compared to WT group (*p* < 0.05). VAChTKD animals exposed to Local 1 compared to WT animals exposed at the same site had an increase in positive cells for NF-KappaB, IL-5, IL-13 (*p *< 0.05). The same increase was observed in those of the VAChTKD Local-2 group (*p* < 0.05).

Comparing the WT animals, we found that there was an increase in IL-17-positive cells in animals exposed to Locals 1 and 2 (*p *< 0.5). There was an increase in IL-17-positive cells in the WT Local-2 group compared to the WT Local-1 group (*p* < 0.05) and an increase in IL-5-positive cells in the WT Local-1 group compared to the WT Local-2 group (*p* < 0.05).

Comparing the VAChT KD animals, we found that there was an increase NF-KappaB- and IL-5-positive cells in the VAChT KD Local-1 group compared to the VAChT KD Local-2 group (*p* < 0.05). There was an increase in IL-17 positive cells in animals exposed to Local 2 compared to other groups (*p *< 0.5) (Table 2).

#### Remodeling

There was an increase in TGF-β-positive cells in VAChT KD animals compared to WT animals (*p* < 0.05).

In summer, there was increase in TGF-β-positive cells in VAChT KD Local-2 animals compared to WT Local-2 group (*p* < 0.05). In winter, there was increase in TGF-β-positive cells in VAChT KD Local-1 animals compared to WT Local-1 group (*p* < 0.05) (Table 2).

### Experimental protocol 2 (Fig. 4)

**Fig. 4:**
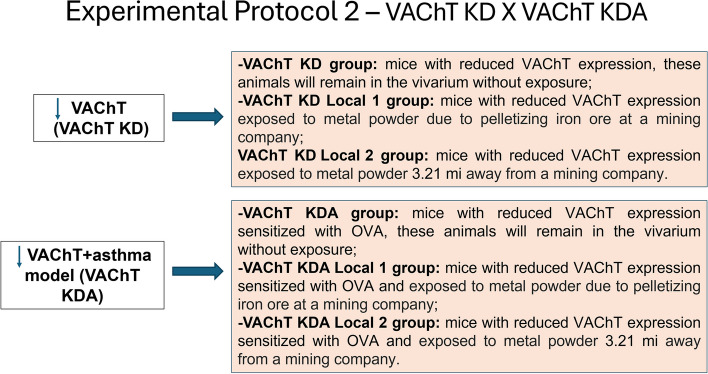
Experimental Protocol 2: Comparative analyses between VAChT KD animals and VAChT KDA animals

#### Hyper-responsiveness

Table 3 demonstrates the analysis of hyperresponsiveness. The animals with decreased VAChT and chronic allergic lung inflammation (VAChT KDA group) showed an increase in the maximal response of %Rrs, %Raw and %Ers compared to animals without chronic allergic lung inflammation (VAChT KD) (*p* < 0.05).

**Table 3:** Analyses of hyperresponsiveness, cholinergic system, inflammation markers, remodeling and oxidative stress (Experimental Protocol 2)

**Hyperresponsiveness**						
** Summer**	**VAChT KD**	**VAChT KD-Local1**	**VAChT KD-Local2**	**VAChT KDA**	**VAChT KDA-Local1**	**VAChT KDA-Local2**
%Rrs	70.5 ± 12.5	**125.2 ± 33.2**^**#**^	50.3 ± 12.0	177.8 ± 36.1^*^	294 ± 104.4	**152.1 ± 47.1**^**#**^
%Ers	17.2 ± 3.7	39.4 ± 4	46.2 ± 7.3^*^	32 ± 3.2^*^	89.2 ± 31.0	67.2 ± 22.9
%Raw	102.9 ± 20.0	177.2 ± 55.7	94.6 ± 29.1	269.0 ± 42.5^*^	152.1 ± 54.0	**265.6 ± 56.8**^**#**^
%Gtis	44.1 ± 10,6	68.8 ± 21.5	23.1 ± 3.7	64.1 ± 12.2	38.9 ± 9.7	**95.7 ± 34.6**^**#**^
%Htis	22.4 ± 4.4	34.2 ± 11.4	58.4 ± 9.3*	27.0 ± 5.9	60.1 ± 19.2	35.4 ± 9.5
** Winter**	**VAChT KD**	**VAChT KD-Local1**	**VAChT KD-Local2**	**VAChT KDA**	**VAChT KDA-Local1**	**VAChT KDA-Local2**
%Rrs	70.6 ± 12.6	79.0 ± 26.4	90.2 ± 31.2	194.8 ± 39.0^*^	144.9 ± 44.4	112.9 ± 14.4
%Ers	21.5 ± 7.6	38.9 ± 3.9	24.8 ± 5.6	32.7.3 ± 3.9	102.0 ± 21.6^**,$^	**81.1 ± 13.0**^**#**^
%Raw	102.8 ± 24.6	81.7 ± 16.0	145.1 ± 51.5	276.6 ± 39.4^*^	176.9 ± 87.6	141.4 ± 22.2
%Gtis	43.0 ± 11,2	24.6 ± 7.8	33.2 ± 13.4	61.5 ± 14.6	118.0 ± 57.0**	91.8 ± 30.1
%Htis	20.4 ± 4.8	35.3 ± 6.3	17.3 ± 6.0	26.4 ± 5.6	96.6 ± 10.3^**,$^	**67.5 ± 17.0**^**#**^
**Cholinergic System**						
** Summer**	**VAChT KD**	**VAChT KD-Local1**	**VAChT KD-Local2**	**VAChT KDA**	**VAChT KDA-Local1**	**VAChT KDA-Local2**
AChR α-7 (cells/10^4^μm2)	1.5 ± 0.3	1.1 ± 0.3	1.0 ± 0.3	8.2 ± 1.2*	15.0 ± 1.8*^,$,+^	**6.3 ± 1.0**^**#**^
** Winter**	**VAChT KD**	**VAChT KD-Local1**	**VAChT KD-Local2**	**VAChT KDA**	**VAChT KDA-Local1**	**VAChT KDA-Local2**
AChR α-7 (cells/10^4^μm2)	1.4 ± 0.4	6.4 ± 1.1*^,#^	3.5 ± 0.8	9.4 ± 0.4*	14.7 ± 1.3**^,$^	**15.1 ± 2.1**^**#,$**^
**Inflammation**						
** Summer**	**VAChT KD**	**VAChT KD-Local1**	**VAChT KD-Local2**	**VAChT KDA**	**VAChT KDA-Local1**	**VAChT KDA-Local2**
Eosinophils (cells/10^4^μm2)	0.8 ± 0.06	2.0 ± 0.1*^,#^	1.2 ± 0.09*	1.5 ± 0.03*	2.7 ± 0.1**^,$^	**3.4 ± 0.1**^**#,$,&**^
NFkappaB (cells/10^4^μm2)	9.5 ± 0.9	6.4 ± 0.8	7.4 ± 1.1	15.5 ± 1.6*	13.3 ± 1.4**	**16.6 ± 1.6**^**#**^
TNF-α (cells/10^4^μm2)	3.7 ± 0.6	2.9 ± 0.6	4.7 ± 0.8	12.8 ± 1.0*	10.08 ± 1.4**	**8.8 ± 1.1**^**#**^
IL-4 (cells/10^4^μm2)	2.6 ± 0.4	2.0 ± 0.5	4.8 ± 0.9^*,**^	8.4 ± 1.0*	10.36 ± 1.5**	**11.6 ± 2.0**^**#**^
IL-5 (cells/10^4^μm^2^)	3.2 ± 3.8	6.3 ± 0.8^*,#^	4.03 ± 0.4	4.6 ± 5.3*	8.05 ± 0.7**^,$^	**9.8 ± 7.1**^**#,$**^
IL-13 (cells/10^4^μm^2^)	2.9 ± 0.3	3.4 ± 0.4	3.3 ± 0.4	3.9 ± 0.5*	5.6 ± 0.5**^,$^	**7.9 ± 0.7**^**#,&,$**^
IL-17 (cells/10^4^μm^2^)	3.2 ± 0.4	2.0 ± 0.5	5.2 ± 0.8*,**	10.8 ± 1.4*	7.4 ± 1.2**	**9.9 ± 1.2 **^**#**^
** Winter**	**VAChT KD**	**VAChT KD-Local1**	**VAChT KD-Local2**	**VAChT KDA**	**VAChT KDA-Local1**	**VAChT KDA-Local2**
Eosinophils (cells/10^4^μm2)	0.8 ± 0.07	1.9 ± 0.07*^,#^	1.2 ± 0.05*	1.4 ± 0.03*	2.7 ± 0.09**^,$^	**3.5 ± 0.1**^**#,$,&**^
NFkappaB (cells/10^4^μm2)	8.9 ± 1.0	16.3 ± 1.7*^,#^	8.9 ± 1.2	14.9 ± 1.7*	21.0 ± 1.4**^,$,+^	**15.0 ± 1.5**^**#**^
TNF-α (cells/10^4^μm2)	3.9 ± 0.7	7.2 ± 1.0*^,#^	2.2 ± 0.5	12.3 ± 1.1*	14.0 ± 1.1**	**11.2 ± 1.2**^**#**^
IL-4 (cells/10^4^μm2)	2.6 ± 0.5	6.0 ± 0.8*^,#^	3.4 ± 0.6	8.0 ± 1.2*	7.6 ± 1.4	**12.6 ± 1.3**^**$,&,#**^
IL-5 (cells/10^4^μm^2^)	3.2 ± 0.3	5.9 ± 5.7*^,#^	3.2 ± 3.9	4.8 ± 0.6*	7.9 ± 0.6**^,$^	**7.5 ± 0.7**^**#,$**^
IL-13 (cells/10^4^μm^2^)	2.6 ± 0.3	3.7 ± 0.4	2.7 ± 0.4	3.7 ± 0.5*	10.8 ± 0.8**^,$,+^	**5.9 ± 0.5**^**$,#**^
IL-17 (cells/10^4^μm^2^)	3.1 ± 0.5	4.2 ± 0.7	3.6 ± 0.7	10.6 ± 1.3*	7.1 ± 0.9**	**10.6 ± 1.5**^**#**^
**Remodeling**						
** Summer**	**VAChT KD**	**VAChT KD-Local1**	**VAChT KD-Local2**	**VAChT KDA**	**VAChT KDA-Local1**	**VAChT KDA-Local2**
MMP-9 (cells/10^4^μm^2^)	1.1 ± 0.3	1.3 ± 0.4	1.1 ± 0.3	5.5 ± 0.6*	5.1 ± 0.9**	**2.8 ± 0.5**^**#**^
TIMP-1 (cells/10^4^μm^2^)	1.1 ± 0.3	1.3 ± 0.4	1.3 ± 0.4	7.5 ± 1.1*	5.7 ± 0.9**	**4.1 ± 0.7**^**#**^
TGFβ (cells/10^4^μm^2^)	4.5 ± 0.9	5.6 ± 1.0	5.8 ± 0.9	18.2 ± 1.7*	16.2 ± 1.6**	**17.4 ± 1.6**^**#**^
Collagen Fibers (%)	0.5 ± 0.1	0.9 ± 0.2	3.9 ± 0.4*,**	1.9 ± 0.4*	1.7 ± 0.3**	**4.3 ± 0.8**^**$,&**^
** Winter**	**VAChT KD**	**VAChT KD-Local1**	**VAChT KD-Local2**	**VAChT KDA**	**VAChT KDA-Local1**	**VAChT KDA-Local2**
MMP-9 (cells/10^4^μm^2^)	1.0 ± 0.3	2.6 ± 0.5*^,#^	0.7 ± 0.3	5.4 ± 0.7*	3.5 ± 0.6	**11.4 ± 1.5**^**#,$,&**^
TIMP-1 (cells/10^4^μm^2^)	1.0 ± 0.3	5.4 ± 0.8*^,#^	1.2 ± 0.5	6.8 ± 1.0*	3.6 ± 0.7	**6.5 ± 1.4**^**#**^
TGFβ (cells/10^4^μm^2^)	3.9 ± 0.8	7.6 ± 1.2	6.3 ± 1.1	18.6 ± 1.9*	15.2 ± 1.4**	**12.9 ± 1.4**^**#**^
Collagen Fibers (%)	0.6 ± 0.1	1.6 ± 0.2	1.5 ± 0.5	2.2 ± 0.4*	3.1 ± 0.5**	**4.9 ± 0.7**^**$,#**^
**Oxidative Stress**						
** Summer**	**VAChT KD**	**VAChT KD-Local1**	**VAChT KD-Local2**	**VAChT KDA**	**VAChT KDA-Local1**	**VAChT KDA-Local2**
No_Ex_	8.5 ± 1.0	11.5 ± 3.1	17.8 ± 6.1	12.5 ± 2.7	30.8 ± 8.3**	25.1 ± 8.2
iNOS (cells/10^4^μm^2^)	1.1 ± 0.4	3.0 ± 1.1	1.9 ± 0.6	5.0 ± 1.3*	9.9 ± 1.5**^,$^	**6.4 ± 0.9**^**#**^
8-PGF-2α (%)	1.0 ± 0.3	3.9 ± 0.6	5.6 ± 1.2*	2.3 ± 0.6*	8.0 ± 1.9	4.5 ± 0.8
** Winter**	**VAChT KD**	**VAChT KD-Local1**	**VAChT KD-Local2**	**VAChT KDA**	**VAChT KDA-Local1**	**VAChT KDA-Local2**
No_Ex_	8.5 ± 1.0	10.1 ± 1.7	9.8 ± 0.9	12.0 ± 2.2	29.2 ± 7.8**	**19.5 ± 4.8**^**#**^
iNOS (cells/10^4^μm^2^)	1.0 ± 0.4	2.6 ± 0.7	1.5 ± 0.6	4.8 ± 1.4*	5.2 ± 0.7**	**5.5 ± 0.9**^**#**^
8-PGF-2α (%)	0.7 ± 0.3	3.1 ± 0.3*	7.6 ± 1.0*,**	1.8 ± 0.4*	5.3 ± 1.7	**22.5 ± 1.3**^**#,$,&**^

^*^p < 0.05 compared to VAChT KD, **p < 0.05 compared to VAChT KD-Local1, ^#^*p* < 0.05 compared to VAChT KD-Local2, ^$^*p* < 0.05 compared to VAChT KDA, ^&^*p* < 0.05 compared to VAChT KDA-Local1, ^+^*p* < 0.05 compared to VAChT KDA-Local2

At Local 1, animals with chronic allergic pulmonary inflammation and VAChT attenuation showed an increase in %Ers and %Htis compared to animals that stayed in the vivarium (*p* < 0.05).

There was an increase in %Ers, Gtis and %Htis in animals with chronic allergic lung inflammation and a decrease in VAChT exposed to local 1 compared to animals without chronic allergic lung inflammation exposed to this local (*p* < 0.05). There was an increase in %Rrs, %Raw, %Gtis and %Htis in animals with chronic allergic lung inflammation and a decrease in VAChT exposed to local 2 compared to animals without chronic allergic lung inflammation exposed to local 2 (*p* < 0.05). 

#### AChR alpha 7 positive cells

Animals with chronic allergic pulmonary inflammation and a decrease in VAChT showed an increase in AChR α-7-positive cells compared to genetically modified animals without chronic allergic pulmonary inflammation (*p* < 0.05), Table 3.

In both stations, there was an increase in AChR α-7-positive cells in the VAChT KDA-Local1 group compared to the VAChT KD-Local1 group (*p* < 0.05). In addition, there was an increase in AChR α-7-positive cells in the VAChT KDA-Local2 group compared to the VAChT KD-Local2 group (*p* < 0.05).

In the comparison analysis between the VAChT KD, VAChT KD-Local1 and VAChT KD-Local2 groups in winter, there was an increase in AChR alpha 7-positive cells in the VAChT KD-Local1 group compared to the VAChT KD-Local2 group (*p* < 0.05). In the comparison analysis between the genetically modified animals in summer, there was an increase in the number of AChR alpha-7-positive cells in the VAChT KDA-Local1 group compared to the VAChT KDA (*p* < 0.05) and VAChT KDA-Local2 groups (*p* < 0.05). In the winter, there was an increase in the number of AChR alpha-7-positive cells in the VAChT KDA-Local1 group and VAChT KDA-Local2 group compared to the VAChT KDA group (*p* < 0.05).

#### Inflammation

For inflammation-related outcomes we evaluated eosinophils, NF-KappaB, TNF-α, IL-4, IL-5, IL-13 and IL 17, its absolute values ​​are shown in the Table 3.

We observed that there was an increase in the number of positive cells in the VAChT KDA group in all analyses compared to the VAChT KD group (*p* < 0.05). For all markers at both stations (summer and winter), we observed that there was an increase in the number of positive cells in the VAChT KDA-Local1 group compared to the VAChT KD-Local1 group (*p* < 0.05) and an increase in the number of positive cells in the VAChT KDA-Local2 group compared to the VAChT KD-Local2 group (*p* < 0.05).

In the comparison analysis between the animals with decreased VAChT, there was an increase in eosinophils, NF-KappaB, TNF-α, IL-4 and IL-5-positive cells in the group exposed to Local 1 compared to animals in the vivarium (*p* < 0.05). The animals exposed to Local 2 showed increased eosinophils and IL-4- and IL-17-positive cells compared to animals in the vivarium (*p* < 0.05).

In summer, there was an increase in eosinophils and IL-5-positive cells in the VAChT KD-Local1 group compared to the VAChT KD-Local2 group (*p* < 0.05), and there was an increase in IL-4- and IL17-positive cells in the VAChT KD-Local2 group compared to the VAChT KD-Local1 group (*p* < 0.05). In the winter, there was an increase in eosinophils and NF-Kappa B-, TNF-α-, IL-4- and IL-5-positive cells in the VAChT KD-Local1 group compared to the VAChT KD-Local2 group (*p* < 0.05).

In both stations, in the comparison analysis between the animals with chronic allergic lung inflammation and VAChT attenuation animals, there was an increase in eosinophils and IL-5- and IL-13-positive cells in the VAChT KDA-Local1 group compared to the VAChT KDA group (*p* < 0.05), an increase in eosinophils, IL-5- and IL-13-positive cells in the VAChT KDA-Local2 group compared to the VAChT KDA group (*p* < 0.05) and an increase in eosinophils in the VAChT KDA-Local2 group compared to the VAChT KDA-Local1 group (*p* < 0.05).

In addition to these differences presented in winter, there was also an increase in NF-KappaB in the VAChT KDA-Local1 group compared to the VAChT KDA group (*p* < 0.05), an increase in NF-KappaB and IL-13 in the VAChT KDA-Local1 group compared to the VAChT KDA-Local2 group (*p* < 0.05), an increase in IL-4 in the VAChT KDA-Local2 group compared to the VAChT KDA group (*p* < 0.05) and an increase in IL-4-positive cells in the VAChT KDA-Local2 group compared to the VAChT KDA-Local1 group (*p* < 0.05).

#### Extracellular matrix remodeling

We demonstrate in Table 3 the results related to extracellular matrix remodeling.

For all markers evaluated, there was an increase in the VAChT KDA group compared to the VAChT KD group (*p* < 0.05). For all markers evaluated in summer, there was an increase in the VAChT KDA-Local1 group compared to the VAChT KD-Local1 group (*p* < 0.5). In winter, we observed the same results in the evaluations of TGF-β and the volume fraction of collagen fibers (*p* < 0.05). In summer, there was an increase of MMP-9-, TIMP-1-, and TGF-β-positive cells in the VAChT KDA-L2 group compared to the VAChT KD-Local2 group (*p* < 0.05); in winter, this was observed in all analyses (*p* < 0,05).

In the comparison analysis between the animals with decreased VAChT in summer, there was an increase in the volume fraction of collagen fibers in the VAChT KD-Local2 group compared to the VAChT KD and VAChT KD-Local1 groups (*P* < 0.05). In the winter, there was an increase in MMP-9- and TIMP-1-positive cells in the VAChT KD-Local1 group compared to the VAChT KD and VAChT KD-Local2 groups (*p* < 0.05).

In the comparison analysis between the animals with chronic allergic lung inflammation and decreased VAChT in summer, there was an increase in the volume fraction of collagen fibers in the VAChT KDA-Local2 group compared to the VAChT KDA group (*p* < 0.05). In the winter, there was an increase in MMP-9 and the volume fraction of collagen fibers in the VAChT KDA-Local2 group compared to VAChT KDA (*p* < 0.05) and an increase in MMP-9 compared to the VAChT KDA-Local1 group (*p* < 0.05).

#### Oxidative stress

We demonstrate in Table 3 the results related to oxidative stress as NO exhaled, iNOS-positive cells and volume fraction of 8-isoPGF-2α.

There was an increase in the values analyzed for iNOS and 8-isoPGF-2α in the VAChT KDA group compared to the VAChT KD group (*p* < 0.05).

There was an increase in iNOS-positive cells in the VAChT KDA-Local1 group compared to the VAChT KD-Local1 group (*p* < 0.5). There was an increase in iNOS-positive positive cells in the VAChT KDA-Local2 group compared to the VAChT KD-Local2 group (*p* < 0.5).

There was an increase in the NO exhaled and the volume fraction of 8-isoPGF-2α in the VAChT KDA-Local1 group compared to the VAChT KD-Local1 group (*p* < 0.05) and the same in the VAChT KDA-Local2 group compared to the VAChT KD-Local2 group (*p* < 0.05).

In the comparison analysis between the VAChT KD, VAChT KD-Local1 and VAChT KD-Local2 groups in both stations there was an increase in the volume fraction of 8-isoPGF-2α in the VAChT KD animals exposed to Local 2 compared to the animals that remained in the vivarium (*p* < 0.05). In winter, there was an increase in the volume fraction of 8-isoPGF-2α in the VAChT KD animals exposed to Local 1 compared to the animals that remained in the vivarium (*p* < 0.05), and there was an increase in the volume fraction of 8-isoPGF-2α in the VAChT KD-Local2 group compared to the VAChT KD-Local1 group (*p* < 0.05).

In the comparison analysis between the VAChT KDA, VAChT KDA-Local1 and VAChT KDA-Local2 groups in summer, there was an increase in iNOS-positive cells in the VAChT KDA-Local1 group compared to the VAChT KDA group (*p* < 0.05). In the winter, there was an increase in the volume fraction of 8-isoPGF-2α in the VAChT KDA-Local2 group compared to the VAChT KDA-Local1 group (*p* < 0.05).

**Fig. 5:**
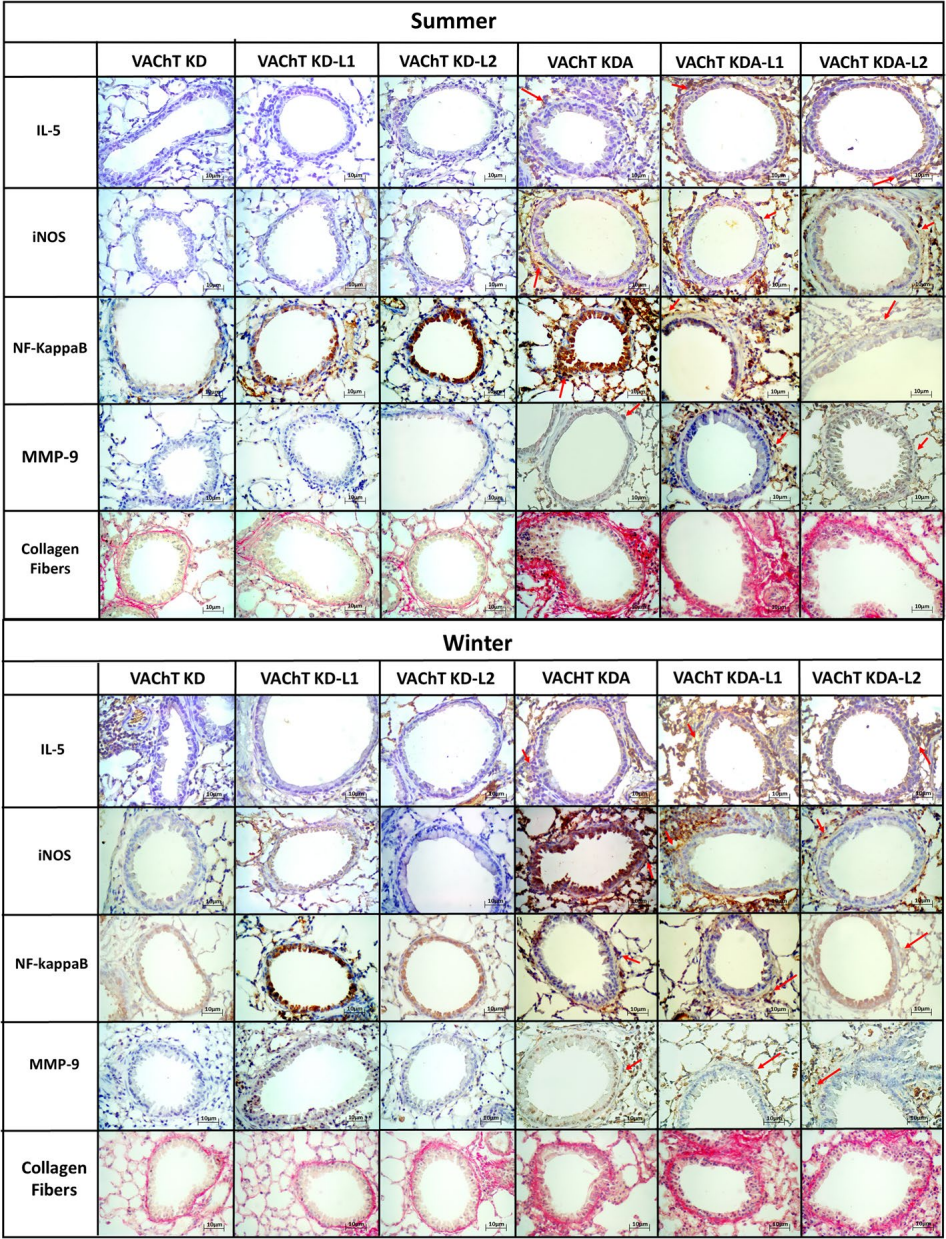
Photographs of analyzed airways. Red arrows demonstrate positive cells

We performed a photo panel of some markers of inflammation, oxidative stress and airway remodeling, it is also possible to qualitatively verify a greater presence of positive cells in animals with chronic allergic pulmonary inflammation and their exacerbation in groups exposed to the sites in both summer and winter. Through some red arrows in the images (Fig. 5). it is possible to identify what would be positive cells, so that the visualization of this increase through the photographs is understandable.

**Fig. 6:**
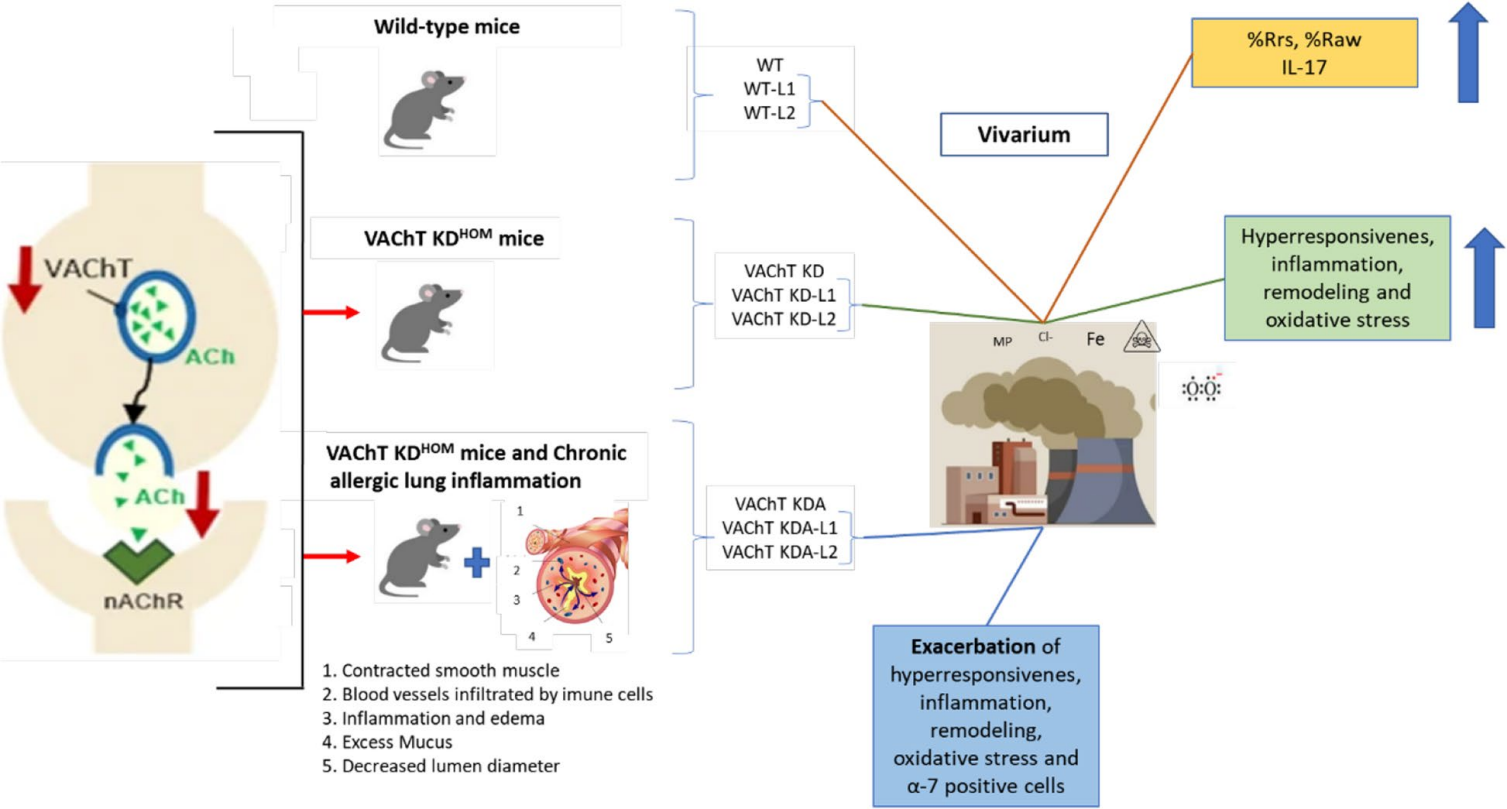
Results summary illustration. Categorization of wild-type and VAChT KD^hom^ animals with and without chronic allergic lung inflammation exposed or not to iron dust pollution and its effects. Adapted image from Santana et al. (2021) [33]

Figure 6 provides a concise overview of the findings from our study, illustrating the effects of VAChT decrease in genetically modified animals exposed to iron dust pollution with and without chronic allergic lung inflammation.

## Discussion

In our study, we observed associations related to ACh and lung inflammation. When VAChT decrease (experimental protocol 1), we observed an increase in NF-kappaB, IL-5 and IL-13 due to a decrease in VAChT compared to wild-type animals. We showed an increase in alpha-7 in VAChT KD with lung inflammation (experimental protocol 2). Notably, this increase was even more pronounced when these mice were exposed to iron dust. The rise in lung α7 nAChR may indeed be linked to their anti-inflammatory properties [34]. These findings shed light on the intricate role of ACh in modulating lung responses and inflammation.

Santana et al. (2019) corroborates our findings showing that IL-13 in VAChT-KD mice were more pronounced than wild type (WT) mice, indicating severe lung alterantions [23]. Pinheiro et al. [34] showed that VAChT deficiency is not unable to raise nicotinic receptor levels to counter lung inflammation, as observed in WT mice. Insufficient ACh for α7 nAChR receptors may contribute to lung inflammation by suppressing the cholinergic anti-inflammatory pathway [34].

During summer animals with VAChT attenuation exposed to Local 1 showed an increase in %Rrs when compared to Local 2, whereas animals in Local 2 showed an increase in %Ers and %Htis when compared to vivarium.

In VAChT animals with lung inflammation, there was an increase in %Rrs and %Raw compared to VAChT-KD without inflammation. Increase in pulmonary resistance is probably due to pulmonary alterations due to remodeling [30].

Regarding cytokine responses, in summer, animals with VAChT attenuation exposed in Local1 exhibited increase IL-5 levels compared to those in the bioterium. We observed that animals that in Local 2 showed an increase in IL-4- and IL-17 compared to those of bioterium and inside mining company. In winter, we observed an increase in NFKappa-B-, TNF-α, IL-4 and IL-5 in animals in Local 1 compared to to the vivarium and Local 2. A previous study evaluating animals with attenuated VAChT expression exposed to diesel exhaust particles also reported increase in IL-4- and IL-13 [23]. Exposure to PM can impact immune regulation, inhalable pollutants trigger innate immune response through increased activation of dendritic cells, lung inflammation, and Th2 immune responsiveness [35]. PM can induce Th1/Th2/Th17 responses [36, 37] and acetylcholine signaling directs dendritic cells towards a Th2 response [38, 39].

The presence of chlorine in Local 2 may explain the IL-17 findings in WT animals. Genraro et al. (2021) showed that chlorine exposure worsens lung function, induces of oxidative stress, promotes mucus production, and contributes to increased inflammation [40].

We observed an increase in all evaluated inflammatory markers (NFKappa-B, TNF-α, IL4, IL-5, IL-13 and IL-17) in animals VAChT with lung inflammation compared to controls. These results are similar to those found on animals with lung inflammation that lack VAChT attenuation [30, 31, 41].

In the summer, there was an increase in IL5- and IL-13 in animals with lung inflammation and attenuation of VAChT exposed to both locals compared to VAChT KDA animals without exposure to pollution. There was increase in IL-13 in VAChT KDA Local-2 compared to exposure to Local 1. In winter, there was an increase in NF-kappaB, IL-13 and IL-17 in the VAChT KDA in Local 1 compared to VAChT KDA animals and an increase in IL-4, IL-5 and IL-13 in VAChT KDA-L2 group compared to VAChT KDA. Local 1 had higher NF-KappaB and IL-13 levels than Local2, and Local 2 had increase IL-4 compared to Local1.

The potentiation of inflammation was observed in eosinophils, IL-4, IL-5, and IL-13 in VAChT animals with lung inflammation in Local 2 compared to those with lung inflammation that remained in vivarium. This effect may be attributed to the high presence of chlorine in Local 2, corroborating the findings of Genaro et al. (2018) [41].

Pollution has been clinically associated with exacerbations of asthma [36] due to stimulation of cells through Toll-like receptors and ROS sensing pathways. This activates NF-KappaB [36, 42]. ROS can disrupt intracellular ion signaling [43]. Intracellular Ca^2+^ influence the airway smooth muscle. PM may increase Ca^2+^ levels in the airways [36].The authors reinforce that composition of PM can vary geographically and temporally [36], so we believe that due to some difference in PM of Local 2, mice presented an increase in IL-5 in summer and IL-4 in winter.

Inhaling urban PM trigger inflammation in airways [37], and high metal concentrations in PM enhance inflammation and airway hyperresponsiveness. This inflammation can be accompanied by release of IL-5, IL-13, IFNγ, and IL-17A [37]. Reference particles such as carbon black, DEPs, and coal fly ash did not produce similar results [36].

Studies showed to exposure to PM2.5 worsened symptoms in rats with allergic rhinitis, leading downregulated of IFN-γ and upregulated of IL-4, IL-13, and eosinophils in nasal fluid [44].

ACh activation via M3 receptors triggers glycogen synthase kinase (GSK)-3 inhibition that suppresses proliferation of airway smooth muscle and promotes remodeling [45]. TGF-β and M2 receptors regulate production of extracellular matrix proteins [46].

We observed in summer that animals with attenuation of VAChT in Local 2 showed an increase in collagen fiber compared to VAChT KD animals in Local 1, and in winter, animals with reduced VAChT in Local 1 showed an increase in MMP-9 and TIMP-1 compared to VAChT KD animals in Local 2. Studies showed that exposure to DEP induced an increase in collagen in mice with attenuation of VAChT, suggesting an effect in airway remodeling [23]. We believe that pollution attenuated VAChT and may generate responses related to remodeling through nicotinic receptors.

Our study revealed a consistent increase in remodeling markers (MMP-9, TIMP-1, TGF-β and collagen) in animals with lung inflammation and VAChT attenuation compared to animals that were not exposed to ovalbumin, these findings align with previous research [20]. Bronchoconstriction induced by methacholine results in increase of smooth muscle myosin, through TGF-β [47–50]. Additionally, bronchoconstriction mediated by acetylcholine (ACh) can result in airway remodeling due to its mechanical effects [19, 51]. Research indicates that not only do airway neurons play a role in transient acute exacerbations, but remodeling of the neuronal architecture also accompanies persistent airway hyperresponsiveness (AHR) [52].

In our study, animals with lung inflammation and with VAChT attenuation in Local 2 exhibitedan increase in MMP9 in winter. Remarkably, even when situated 3.21 miles away from themining company, these animals showed elevates collagen levels in both seasons. Studies recommended that concentrations of motor vehicle emissions, such as ultrafine PM and carbon black particulates decrease significantly within 300 m [53] and distances within 300–500 m of highways affected human health [54]. ROS and mechanical stress have been identified as factors that can trigger proliferation of ASM [55].

Meteorological conditions influence dispersion and accumulation of pollutants [56–58]. Liu and Johnson (2002) described that air pollution is generally associated with factors such as temperature, relative humidity, wind speed and direction, among others [57]. The occurrence of rainfall and the increase in wind speed contribute to the dispersion and dilution of pollutants and, consequently, to the reduction of their concentration [57]. It is noteworthy that in the summer there are greater volumes of rain than in winter. The trend is that for pollutants such as PM10, the concentration is lower in the summer period [59].

Animals with VAChT attenuation in Local 1 and Local 2 showed an increase in 8-PGF-2α when compared to controls. Animals in Local 2 showed an increase in 8-PGF-2α when compared to Local 1. Researchers have shown that even in healthy lungs, PM2.5 is associated with elevated levels of oxidative stress and presence of pro-inflammatory biomarkers [60]. Organic compounds found in PM can transfer electrons to O_2_ molecules, forming superoxide free radicals. Transition metals can transfer electrons to form superoxide and hydrogen peroxide [36].

PM cytogenotoxic action is attributed to metallic components, such as iron and transition metals, which can induce the formation of ROS [36, 61, 62]. Iron particles stimulate the production of hydroxyl radicals [61, 63]. ROS can be generated from the surface of particles where polycyclic aromatic hydrocarbons (PAHs) and nitro-PAHs are absorbed, with exception of transition metals (iron, copper, chromium and vanadium), which catalyze Fenton reaction generate highly reactive hydroxyl radicals capable of inducing oxidative damage to DNA [63]. Given that exposure sites often contain large amounts of iron (due to the pelleting process), these effects are particularly relevant.

Seaton et al. (2005) demonstrated that dust on London underground railway had cytotoxic and inflammatory effects at high doses, consistent with its largely iron oxide composition [62]. Soluble metals in inhaled particles, such as Fe, nickel (Ni), vanadium (V), cobalt (Co), copper (Cu) and Cr, are associated with increased ROS production, leading to cellular oxidative stress [64–66].

In our study, animals with lung inflammation and VAChT attenuation exhibited increased levels os iNOS and 8-PGF-2α compared to controls animals.. In summer, there was an increase in iNOS in animals with lung inflammation in Local 1 compared to animals with lung inflammation in vivarium. In winter, there was an increase in 8-PGF-2α in Local 2 in relation to animals in vivarium and those in Local 1. As observed in isoprostane there was the same as that of healthy animals with lung inflammation. We hypothesize that a specific pollutant in Local 2 directly contributes to isoprostane-dependent oxidative stress pathways. NF-κB can be generate oxidative stress, leading to regulation of pro-inflammatory cytokines, enzymes, and adhesion molecules [67]. Inflammatory processes associated with asthma have a dynamic relationship with increased levels of ROS [68]. These findings highlight the interplay between oxidative stress and airway inflammation.

By analyzing the change in these markers when animals with attenuation of VAChT and asthma were exposed to pollution, we can observe the impact of this exposure on health. Asthma exacerbated by pollution is a clinical challenge, and treatment aims to control symptoms and reduce airway inflammation. Strategies for managing pollution-exacerbated asthma include educating the public, reducing pollution, air quality regulation, monitoring hospitalizations, and providing evidence-based counseling. Healthcare staff need reliable local data on air pollution and patients should be asked about their work environments and exposure locations, access to quality educational materials is also critical for the population [69, 70].

## Conclusions

We provide evidence that reduced cholinergic signaling exacerbates lung inflammation in a model of chronic allergic lung inflammation. Furthermore, when combined with pollution exposure, this effect it can be amplified, impacting responses related to inflammation, oxidative stress, and remodeling.

Cholinergic deficiency can worsen lung changes caused by exposure to pollution. A functional cholinergic system is necessary to protect the lungs from the effects of air pollution.

No prior studies have evaluated the impact of the cholinergic system on animals with chronic allergic pulmonary inflammation exposed to iron dust pollution. There are few studies on the cholinergic system regarding the inflammatory responses of each pollutant and its pulmonary effect. Given the diversity of pollutants, caution must be taken in assuming that all pollutants will act in a similar way.

Our research underscores the importance of cholinergic pathways and their modulation of air pollution. Further investigations will elucidate other specific mechanisms.

### Supplementary Information

Supplementary Material 1. Data regarding immunohistochemistry describing markers, dilutions, secondary antibodies, AQ and specifications.

## Data Availability

No datasets were generated or analysed during the current study.
