# Correction: Effect of VAChT reduction on lung alterations induced by exposure to iron particles in an asthma model

**DOI:** 10.1186/s12950-024-00401-1

**Published:** 2024-07-30

**Authors:** Tabata Maruyama dos Santos, Renato Fraga Righetti, Leandro do Nascimento Camargo, Edna Aparecida Leick, Silvia Fukuzaki, Elaine Cristina de Campos, Thiago Tafarel Galli, Beatriz Mangueira Saraiva-Romanholo, Luana Laura Sales da Silva, Jéssica Anastácia Silva Barbosa, Juliana Morelli Lopes Gonçalves João, Carla Máximo Prado, Bianca Goulart de Rezende, Christine Laure Marie Bourotte, Fernanda Degobbi Tenorio Quirino dos Santos Lopes, Milton de Arruda Martins, Isabela M. Bensenor, João Vitor de Oliveira Cirillo, Suellen Karoline Moreira Bezerra, Fabio José Alencar Silva, Marcela Souza Lima Paulo, Paulo A. Lotufo, Iolanda de Fátima Lopes Calvo Tibério

**Affiliations:** 1 Faculdade de Medicina FMUSP, Universidade de Sao Paulo, São Paulo, SP Brazil; 2 Hospital Sírio Libanês, São Paulo, SP Brazil; 3 Hospital Alemão Oswaldo Cruz, São Paulo, Brazil; 4 University City of São Paulo (UNICID), São Paulo, Brazil; 5 Department of Biosciences, Universidade Federal de São Paulo, Santos, Brazil; 6 Escola Superior de Ciências da Santa Casa de Misericórdia de Vitória, Vitória, Brazil; 7 Instituto de Geociências, Universidade de São Paulo, São Paulo, Brasil

**Correction to: Journal of Inflammation (2024) 21:24**

10.1186/s12950-024-00399-6

Following the publication of the Original Article, the authors identified that the PDF version contained incorrect author names due to typesetting errors. Moreover, the authors reported that the names of Milton Arruda Martins and Iolanda Fátima Lopes Calvo Tibério were missing particles.

**Incorrect**:

Silvia Fukusaki

Fernanda Degobbi Tenorio Quirino Santos dos Lopes

Milton Arruda Martins

Isabela Judith Martins Bensenor

Paulo Andrade Lotufo

Iolanda Fátima Lopes Calvo Tibério

**Correct**:

Silvia Fukuzaki

Fernanda Degobbi Tenorio Quirino dos Santos Lopes

Milton de Arruda Martins

Isabela M. Bensenor

Paulo A. Lotufo

Iolanda de Fátima Lopes Calvo Tibério

The author group has been updated above and the Original Article has been corrected.

